# Deep Learning-Based Algorithm for the Classification of Left Ventricle Segments by Hypertrophy Severity

**DOI:** 10.3390/jimaging11070244

**Published:** 2025-07-20

**Authors:** Wafa Baccouch, Bilel Hasnaoui, Narjes Benameur, Abderrazak Jemai, Dhaker Lahidheb, Salam Labidi

**Affiliations:** 1 Research Laboratory of Biophysics and Medical Technologies LR13ES07, Higher Institute of Medical Technologies of Tunis, University of Tunis El Manar, Tunis 1006, Tunisia; narjes.benameur@istmt.utm.tn (N.B.); salam.labidi@istmt.utm.tn (S.L.); 2 SERCOM-Lab, Tunisia Polytechnic School, EPT, Carthage University, Tunis 2078, Tunisia; bilel.hasnaoui@ept.ucar.tn; 3 SERCOM-Lab, Tunisia Polytechnic School, INSAT, Carthage University, Tunis 1080, Tunisia; abderrazak.jemai@insat.rnu.tn; 4 Faculty of Medicine of Tunis, University of Tunis El Manar, Tunis 1007, Tunisia; dhaker.lahidheb.cardio@gmail.com; 5 Department of Cardiology, Military Hospital of Tunis, Tunis 1008, Tunisia

**Keywords:** left ventricle hypertrophy, automatic quantification, CNN classification, regional wall thickness, cine-MRI

## Abstract

In clinical practice, left ventricle hypertrophy (LVH) continues to pose a considerable challenge, highlighting the need for more reliable diagnostic approaches. This study aims to propose an automated framework for the quantification of LVH extent and the classification of myocardial segments according to hypertrophy severity using a deep learning-based algorithm. The proposed method was validated on 133 subjects, including both healthy individuals and patients with LVH. The process starts with automatic LV segmentation using U-Net and the segmentation of the left ventricle cavity based on the American Heart Association (AHA) standards, followed by the division of each segment into three equal sub-segments. Then, an automated quantification of regional wall thickness (RWT) was performed. Finally, a convolutional neural network (CNN) was developed to classify each myocardial sub-segment according to hypertrophy severity. The proposed approach demonstrates strong performance in contour segmentation, achieving a Dice Similarity Coefficient (DSC) of 98.47% and a Hausdorff Distance (HD) of 6.345 ± 3.5 mm. For thickness quantification, it reaches a minimal mean absolute error (MAE) of 1.01 ± 1.16. Regarding segment classification, it achieves competitive performance metrics compared to state-of-the-art methods with an accuracy of 98.19%, a precision of 98.27%, a recall of 99.13%, and an F1-score of 98.7%. The obtained results confirm the high performance of the proposed method and highlight its clinical utility in accurately assessing and classifying cardiac hypertrophy. This approach provides valuable insights that can guide clinical decision-making and improve patient management strategies.

## 1. Introduction

### 1.1. Motivation and Background

Cardiovascular diseases (CVD) stand as the primary global cause of mortality, representing one of the most significant health challenges worldwide [1,2]. Left ventricle hypertrophy (LVH) is the most prevalent genetic heart disorder, which is characterized by excessive thickening of the LV muscle. Impacting around 15% to 20% of the population in both developed and developing countries, it represents a significant health concern [3]. LVH is an independent predictor of future cardiovascular events, including coronary heart disease, heart failure, and stroke, regardless of its underlying etiology. This means that regardless of whether LVH arises from hypertension, obesity, or genetic factors, its presence alone significantly elevates the risk of serious cardiovascular complications such as arrhythmias and sudden cardiac arrest.

Recognizing LVH as a critical risk marker underscores the importance of early detection and proactive management to prevent adverse cardiovascular outcomes. Therefore, comprehensive screening and targeted interventions for individuals with LVH are essential components of effective cardiovascular disease prevention strategies [4]. In clinical practice, echocardiography is the first-line examination for the assessment of LVH. However, this imaging modality is characterized by a limited ability to differentiate between various LVH phenotypes and other forms of myocardial thickening. In such cases, cardiac magnetic resonance imaging (CMRI), as a multiparametric and non-invasive imaging modality, is performed to address the limitations of echocardiography [5,6]. Identifying LVH requires precise and reliable diagnostic parameters. While left ventricle mass (LVM) provides a global assessment of heart muscle hypertrophy, it often falls short in detecting regional variations accurately. In some cases, a normal LVM may not accurately reflect the presence of segmental hypertrophy within the LV. This discrepancy occurs when certain myocardial segments are hypertrophied while others are thinned, resulting in an overall normal LVM measurement [7].

This limitation makes the LVM an unreliable parameter for detailed regional analysis. In response to this issue, efforts are directed towards evaluating the regional wall thickness (RWT) of the myocardium. This parameter serves as a crucial marker for several cardiac conditions including ischemic heart disease and heart failure. It has been widely used in studies to assess not only structural abnormalities but also the extent of myocardial damage and the evaluation of myocardial viability [8]. Motivated by these limitations and the clinical need for a more accurate method to quantify LVH, this work leverages RWT as a key parameter to investigate regional hypertrophy. We focus on localized variations in myocardial wall thickness (MWT) and examine their clinical implications in terms of disease progression and diagnostic precision. This approach offers a more refined and segment-specific analysis, enabling the detection of hypertrophied regions that might otherwise be missed by global indices such as LVM. By capturing even subtle structural changes, it enhances diagnostic accuracy and provides valuable insights into the underlying pathophysiology of LV remodeling [9].

### 1.2. Objectives and Contributions

This research paper aims to introduce an automated approach utilizing a convolutional neural network (CNN) for the detection and regional quantification of the LVH and the classification of LV myocardial segments according to hypertrophy severity based on RWT quantification. Key contributions of this study include the following:

(a) A fully automated pipeline for myocardial thickness extraction has been developed. This process involves intensity normalization, automated delineation of endocardial and epicardial contours using a validated U-Net architecture, and anatomical segmentation of the left ventricle into 17 standard regions. Each region was subsequently partitioned into three sub-segments to enable a more detailed regional assessment. Myocardial thickness was then computed automatically for each sub-segment and adopted as the primary indicator of hypertrophy. This parameter offers higher sensitivity to early and localized structural changes compared to global indices such as LVM, which may fail to detect focal abnormalities in routine clinical evaluations.

(b) A dedicated CNN architecture has been developed to automatically classify myocardial sub-segments according to hypertrophy severity, using four clinically defined categories: normal, mild, moderate, and severe. Unlike most existing approaches, which primarily explore the underlying causes of hypertrophy, this work emphasizes the precise quantification of its extent at a regional scale. The architecture incorporates average pooling layers, which were deliberately chosen to preserve subtle structural variations in myocardial thickness, thereby improving the model’s sensitivity to fine-grained severity patterns. The clinical relevance of the proposed method was confirmed through a comparative analysis between automated classifications based on bull’s-eye diagrams and expert visual assessments.

(c) An original dataset of myocardial sub-segments annotated by hypertrophy severity was compiled from cine-MRI scans collected at two independent clinical institutions: the Military Hospital and the Pasteur Clinic of Tunis. To minimize scanner-related biases and ensure consistency across imaging systems, all images were preprocessed using min-max intensity normalization. For each patient, the LV was automatically subdivided into 49 sub-segments, which were initially annotated by an experienced radiologist and subsequently reviewed by a senior cardiologist. Each sub-segment was classified into one of four clinically meaningful categories: normal, mild, moderate, or severe hypertrophy. To the best of our knowledge, this is the first dataset to offer such fine-grained, expert-validated annotation of hypertrophy severity at the sub-segmental level, thus representing a valuable asset for advancing research in cardiac imaging and deep learning-based diagnosis.

## 2. Related Research

Given the importance of early management of LVH, various methods have been proposed in the literature to improve the diagnosis of this heart disease. For instance, Maanja et al. [10] aimed to discern the clinical characteristics that correlate with true and false positives in an Electrocardiogram (ECG) based on artificial intelligence (AI) algorithm. This investigation was intended to enhance the algorithm’s accuracy in detecting hypertrophic cardiomyopathy (HCM) and to refine its application in clinical settings. In another study [11], researchers developed multivariable logistic scores using both conventional and advanced ECG measures to diagnose increased LVM and global wall thickness. Advanced ECG scores outperformed conventional criteria, indicating distinct electrophysiological signatures for each condition. Furthermore, Kokubo et al. [12] developed deep learning (CNN) and machine learning models (logistic regression and random forest) to detect LV dilation and LVH using ECG and echocardiographic data. Their findings revealed that for detecting LVH, the Area Under the Receiver Operating Characteristic (AUROC) was notably higher with deep learning methods compared to machine learning approaches. Li et al. [13] constructed a diagnostic model for HCM using a combination of a random decision forest and an artificial neural network. This model utilized 40 genes identified from both the training and verification sets to predict the disease, to enable early detection and intervention. Budai et al. [3] devised automated techniques utilizing a three-dimensional variant of ResNet for identifying LVH from CMR images captured in both short-axis and long-axis views. The network outputs a decision regarding the presence or absence of hypertrophy aiming to boost diagnostic precision. Furthermore, Duffy et al. [14] introduced a deep learning framework that employed a modified DeepLabv3 architecture, trained on parasternal long-axis images, to minimize weighted mean square error loss. The objective was to accurately quantify LVH and predict the underlying cause of increased LV wall thickness. Soto et al. [15] proposed a multimodal deep learning model to enhance the diagnostic precision of LVH by determining the etiology of this cardiac genetic disease using a late average fusion neural network. In addition, Jian et al. [16] proposed a CNN model under the TensorFlow framework to aid in the diagnosis of LVH through posterior wall thickness measurement. Hwang et al. [17] developed a convolutional neural network long short-term memory (CNN LSTM) for the differential diagnosis of three common LVH etiologies (hypertensive heart disease, HCM, and light chain cardiac amyloidosis).

In a recent study [18], the authors applied machine learning techniques including decision trees, random forests, and support vector machines to assess the pretest probability of identifying the origin of LVH. This work specifically focused on predicting whether left ventricular hypertrophy was due to hypertension. To improve LVH prediction, several studies, such as those in [19,20,21], trained machine and deep learning models, including logistic regression, random forest, LGBM, ResNet, and CNN using features derived from ECGs, such as R-wave and S-wave amplitudes, heart rates, time intervals, and electrical axes. The obtained results proved that machine learning models surpass traditional ECG criteria in classifying and predicting LVH and outperformed CNN models trained directly on ECG signals. In related research, Khurshid et al. [22] developed a deep learning model using ECG data to estimate LV mass derived from cardiac magnetic resonance (CMR), aiming to evaluate their effectiveness in detecting LVH. Zhou et al. [23] proposed a deep learning model to improve mutation-risk prediction of HCM by extracting CMR morphological features. This work aimed to classify the HCM genotypes, based on a nonenhanced four-chamber view of cine images. In a different context, Jesús M. et al. [24] investigated an uncommon cardiomyopathy known as left ventricular non-compaction (LVNC), which is characterized by a thick and spongy left ventricular wall due to hypertrophy of trabeculae. The objective was to measure the percentage of the trabecular volume using a deep learning-based method (2D-U-Net). Most of the proposed studies have shown that deep learning models are effective in diagnosing LVH, either by detecting its presence or by differentiating the causes leading to this ventricular condition, with high accuracy. However, these models still require refinement for routine clinical integration. Moreover, accurately assessing the extent and severity of LVH remains a challenging task in clinical practice. Overcoming this challenge is essential for guiding treatment strategies and estimating prognosis, which is the primary focus of this paper.

## 3. Materials and Methods

### 3.1. Study Design

All procedures carried out in this study adhered to the ethical standards of the institutional research committee and conformed to the principles of the 1964 Helsinki Declaration and its subsequent amendments. As the study involved only anonymized imaging data without any patient identifiers or personal information, formal consent was not required. This study aimed to design an automatic framework for detecting and quantifying LVH, while incorporating normal cases to ensure balanced and representative data. The methodology relies on a structured pipeline composed of multiple steps, aimed at extracting detailed anatomical features and enabling the classification of myocardial hypertrophy at the sub-segmental level.

First, cine cardiac MRI images were preprocessed through a dedicated pipeline involving intensity normalization and automatic segmentation of the left ventricular endocardial and epicardial contours.

Second, the RWT was automatically quantified at 20-degree intervals for the basal and mid-ventricular slices, and at 30-degree intervals for the apical slice, enabling a detailed spatial mapping of myocardial thickness. These RWT values were used as input features for a CNN trained to classify each sub-segment into one of four hypertrophy categories: healthy, mild, moderate, or severe. This method introduces a novel 49-sub-segment model that serves as a diagnostic aid, which is visually represented through a bull’s-eye diagram.

**Figure 1:**
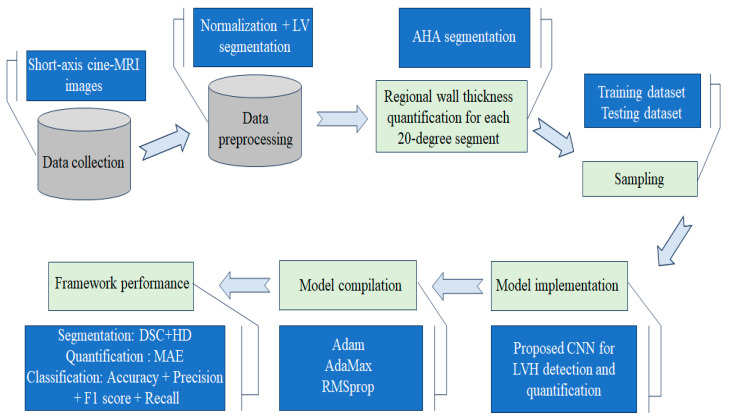
The overall design of the proposed framework for automatic detection, quantification, and classification of LVH.

In the final step, the proposed method was clinically validated, confirming its effectiveness in providing a detailed and intuitive spatial assessment of myocardial hypertrophy severity. The overall architecture and workflow of the proposed framework for early detection and quantification of LVH are summarized in Figure 1.

### 3.2. Study Population and CMR Acquisition

Our dataset was retrospectively collected from the Military Hospital and the Pasteur Clinic of Tunis. After eliminating patients with suboptimal image quality, we investigated 133 patients. We included 87 patients diagnosed with LVH, ranging in age from 23 to 67 years (49 males and 38 females). Additionally, we examined 46 healthy subjects (NOR) with no known cardiovascular diseases, aged between 19 and 45 years (19 males and 27 females). Cine-MR images were acquired in breath-hold in short-axis orientation using SSFP and FLASH sequences with retrospective ECG gating on two MRI scanners with different magnetic field strengths: a 1.5 Tesla from GE Medical Systems and a 3.0 Tesla from Siemens Medical Solutions. For each subject, a cine-MRI sequence comprising 20 to 40 frames was acquired to cover the entire LV, resulting in a total of over 3325 images. The acquisition parameters were configured as follows. For the 3 Tesla MRI machine: repetition time (TR): 2.8 msec, echo time (TE): 1.4 msec, slice thickness: 7 mm, section gap: 3 mm, and field of view (FOV): 300 × 300 mm^2^. For the 1.5 Tesla MRI machine: TR = 4 ms, TE = 2 ms, slice thickness: 8 mm, no gap, and FOV: 320 × 320 mm^2^. For each patient, expert clinician reference segmentations of the endocardial and epicardial contours were provided, along with additional information such as age, weight, height, and the systolic and diastolic phases.

### 3.3. Data Preprocessing

#### 3.3.1. Min-Max Normalization

Our database was collected using MRI machines with varying characteristics, leading to differences in image properties such as spatial resolution, contrast, and signal-to-noise ratio. This diversity of sources and MRI equipment presents an additional challenge when handling the dataset using neural networks. Hence, conducting a normalization step becomes imperative to ensure consistency and high-quality outcomes. In this context, we have opted for the “min-max normalization”, which consists of adjusting the data scale to fit within the range [0, 1]. The aim of constraining the interval in this way is to minimize the range of variation in a feature’s values, thereby reducing the impact of outliers, which are data points that deviate markedly from the rest of the dataset. Normalization is applied using the following formula [25]:

(1) $$X_{Normalized}=\frac{X-X_{min}}{X_{max}-X_{min}},$$

where X is the value of the feature that we seek to normalize, X _min_ is the smallest value observed for the feature X, X _max_ is the largest value observed for the feature X, and X _Normalized_ is the new normalized value of X. Using min-max normalization, the pixel values of the black background are brought close to 0, while the pixel values of the structures of interest are highlighted within the [0, 1] range. This enhances the visualization of important clinical features while preserving contrast nuances. The major advantage of this type of normalization is the preservation of relative contrast between structures in the image, which is crucial for the analysis and visualization of medical images in general, and cine-MRI images in particular. This means that the contrast relationships between different parts of the image remain intact despite the normalization.

#### 3.3.2. Automatic Segmentation of Short-Axis Cine-MR Images Using Deep Learning

In our study, the segmentation of the LV contours is particularly important for identifying regional variations in myocardial thickness, which is essential for accurate diagnosis and proper assessment of the severity of LVH. The significance of this step becomes particularly apparent in cases of asymmetric LVH, where myocardial thickening is not evenly distributed. In clinical practice, radiology experts manually segment cine-MRI images of the LV, which is a time-consuming process that is prone to inter- and intra-observer variability. This subjectivity can lead to significant differences in myocardial thickness measurements, affecting the accuracy of diagnosis and patient follow-up. To overcome these challenges, we propose using an encoder–decoder neural network model to automatically extract the LV borders, based on our previous research [26].

The segmentation task was carried out using a convolutional neural network based on the original U-Net architecture introduced by Ronneberger et al. [27], which includes five encoding and decoding blocks. Starting from this standard configuration, we explored alternative network depths with four and three blocks to determine the most appropriate architecture for our dataset. The goal was to identify a model structure that balances representational capacity and generalization without overfitting, given the modest size of our dataset.

To further refine the segmentation process, we applied average pooling operations to produce smoother segmentation maps and incorporated zero-padding to ensure dimensional consistency between input and output images. The network was trained using cine-MRI images from 70 patients, with a fixed learning rate (LR = 10^−2^), the Adam optimizer, and a mini-batch size of 4. We experimented with training durations of 10, 30, 50, and 80 epochs, which were sufficient for convergence in our setup. The full training process required approximately 95 min. Validation and testing were performed on independent datasets comprising 30 and 33 patients, respectively. The prediction time per image was approximately 2 s.

### 3.4. Automatic Regional Wall Thickness Quantification

In clinical practice, MWT is typically evaluated at two specific phases of the cardiac cycle (end-diastole and end-systole) due to time constraints. While this approach provides a global assessment of myocardial thickness, it may fail to capture localized abnormalities. Indeed, regional variations in wall thickness may be overlooked, limiting the sensitivity of such evaluations, particularly in conditions such as HCM, where hypertrophy may affect only portions of a given region. To achieve more precise and regionalized analysis, radiologists commonly rely on the standardized segmentation of the left ventricle (LV) into 17 myocardial segments, as defined by the American Heart Association (AHA). This segmentation divides the basal and mid-cavity levels into six equal segments of 60°, and the apical level into four segments of 90°.

However, even with this segmentation, certain morphological variations may remain undetected. For example, hypertrophy can affect only part of a segment, while another portion may appear normal or even thinned. Such intra-segmental heterogeneity may lead to inaccurate assessments and overlooked abnormalities. Additionally, in clinical routine, the segmentation of the LV cavity, according to AHA standards, is often performed semi-automatically. The radiologist manually selects key anatomical landmarks: the center of the LV cavity, and the upper and lower intersection points between the LV and right ventricle (RV). These reference points are then used by the post-processing software installed on the scanner console to generate the 17 myocardial segments.

To address these limitations, we developed a fully automatic method that replicates and extends the AHA segmentation based on anatomical landmarks extracted directly from cine-MRI images. Building on this process, we proposed an enhanced approach by subdividing each of the 17 AHA segments into three equal angular sub-segments, thereby improving spatial granularity, except at the apex, where the absence of a left ventricular cavity precludes such division.

This results in an angular resolution of 20° for the basal and mid-cavity regions (originally 60°) and 30° for the apical regions (originally 90°). Consequently, a total of 49 myocardial regions is obtained: 18 for the basal cavity, 18 for the mid-cavity, 12 for the apical cavity, and 1 for the apex. This decision was made in collaboration with two experienced radiologists and a cardiologist, who validated the clinical relevance and interpretability of this level of granularity.

Dividing each segment into three parts reflects an optimal balance. Using four or five subdivisions would yield angular spans of 15° or 12° (basal/mid-cavity) and 22.5° or 18° (apical), which would exceed both the resolution limits of cine-MRI and the interpretive capacity of routine clinical analysis. Moreover, increasing the number of subdivisions would raise the computational cost and complexity without providing proportional diagnostic benefit. In summary, the choice of three sub-segments per original AHA segment was guided by anatomical rationale, clinical expertise, and technical feasibility. It offers a detailed yet robust approach for detecting subtle regional variations in myocardial thickness, improving the precision of hypertrophy assessment.

**Figure 2:**
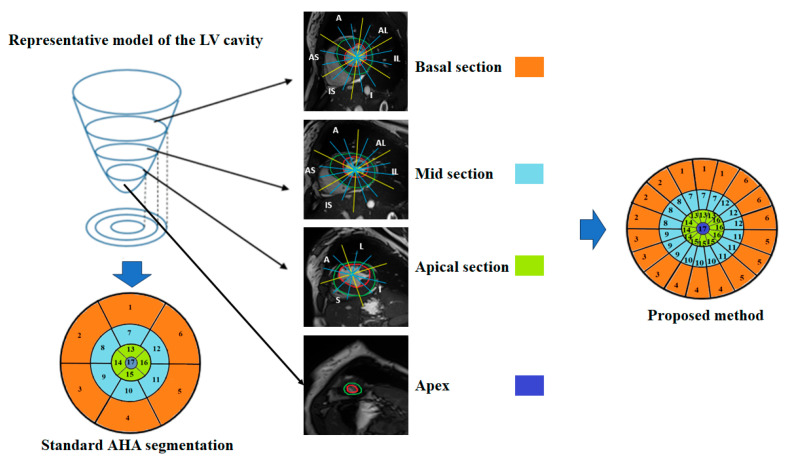
Proposed method for automatic quantification of regional wall thickness.

Finally, for each of the 49 sub-segments, myocardial thickness was computed by measuring the distance between the endocardial and epicardial contours at each angular location using a custom Python algorithm (version 3.6). This fine-grained analysis enabled the detection of subtle intra-segmental variations and improved the accuracy of hypertrophy quantification in both normal and pathological cases. The principle of the proposed method is illustrated in Figure 2.

### 3.5. Proposed Convolutional Neural Network for LVH Detection and Quantification

#### 3.5.1. Ground Truth and Data Sampling

In this section, we provide a detailed description of the CNN we have proposed for classifying LVH based on its severity. Prior to exploring the technical aspects, it is important to note that our database comprises more than 3325 images. Since each patient is represented by 49 myocardial segments, we are analyzing a total of over 6517 myocardial sub-segments. Following discussions with the two radiologists and the cardiologist involved in the study, and based on the quantified regional MWT, all segments were classified into four distinct categories reflecting the severity of LVH. Each group was then assigned a corresponding score as follows:

- Score 0 was assigned to healthy myocardial sub-segments characterized by a wall thickness < 15 mm.
- Score 1 was assigned to sub-segments with mild hypertrophy characterized by a wall thickness in the range [15 mm, 20 mm].
- Score 2 was assigned to sub-segments with moderate hypertrophy characterized by a wall thickness in the range [20 mm, 25 mm].
- Score 3 was assigned to sub-segments with severe hypertrophy characterized by a wall thickness > 25 mm.

This classification is considered the ground truth for our neural network. The preprocessed dataset is then divided into two parts: 70% for the training set, 10% for the validation set, and 20% for the testing set.

#### 3.5.2. Detailed CNN Architecture

**Figure 3:**
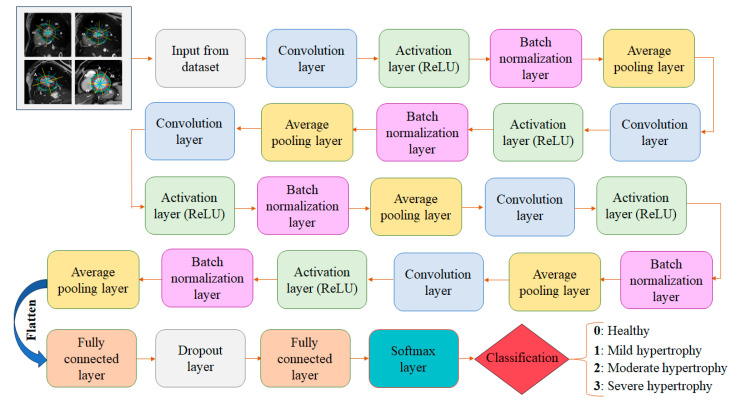
Proposed CNN architecture for myocardial sub-segments classification based on hypertrophy severity.

In this section, we present the architecture of the CNN model designed for the classification of myocardial sub-segments based on the hypertrophy severity. The proposed CNN model is tailored to effectively capture spatial features and subtle variations in MWT, which are critical for accurate classification. The network is composed of 25 layers as described in Figure 3:

- The input layer: The initial layer receives the preprocessed dataset consisting of cine-MRI images acquired in the short-axis orientation. Each image has a size of 256 ×256 and the preprocessing includes several key steps: normalization to standardize pixel intensity values, segmentation of the LV contours, and the division of each myocardial segment into three equal sub-segments.
- Five convolutional layers: Convolutional layers are key components of a CNN responsible for automatically learning and extracting features from the dataset. These layers consist of applying convolutional filters (kernels) to the input images to create feature maps. These filters slide over the image, performing element-wise multiplication and summing the results to produce a single value in the feature map. In our case, with 2D input images and 2D filters, the convolution operation can be mathematically expressed as follows [28]:

(2) $$S\left(i,j\right)=\left(K\;*\;I\right)\left(i,j\right)=\sum_{m}\sum_{n}I\left(i+m\cdot j+n\right)\;*\;K\left(m,n\right)$$

where S(i, j) denotes the value at position (i, j) in the output feature map, I represents the input image, and K is the filter (or kernel). The summation is performed over all indices m and n corresponding to the dimensions of the filter.

- Five activation layers (ReLU): The output of the convolutional layers serves as the input to the activation layers. These layers introduce nonlinearity into the network, allowing it to learn complex patterns and relationships in the data. ReLU activates neurons by outputting the input value directly if it is positive, or zero otherwise. Mathematically, the ReLU function is expressed by the following equation [26].

(3) $$ReLU\left(x\right)=max(0,x)$$

- Five batch normalization layers: These are applied to improve the training and performance of the proposed neural network. By modifying and scaling the activations, it normalizes the output of an earlier activation layer. This stabilizes and accelerates the learning process.
- Five average pooling layers: These layers are integrated into our CNN architecture to reduce spatial resolution while preserving relevant structural information from the feature maps. Although max pooling is commonly used for its ability to emphasize dominant activations, we explored both pooling strategies during the hyperparameter tuning phase to determine which would better preserve clinically meaningful features, particularly in the context of classifying myocardial segments by hypertrophy severity. The results of this ablation study are presented in the Results section (Section 4.1). Based on the outcomes, we selected average pooling for the final architecture, as it consistently yielded slightly superior performance across all evaluation metrics. This choice reflects the specific needs of our task: capturing subtle but clinically relevant differences in myocardial morphology, which may be disregarded due to the selective nature of max pooling that emphasizes only the strongest activations. The average pooling operation computes the mean of the values within a 2 × 2 window and can be mathematically defined as follows [29]:

(4) $$S_{j}=\frac{1}{\left|R_{j}\right|}\sum_{a_{i}\epsilon R_{j}}a_{i},\;\;\;\;\;\;\;I\;=\;1,\;2,\;\ldots |R_{j}|$$

- Two fully connected layers (FC): Each neuron is connected to every neuron in the preceding layer. The FC layers combine and integrate the spatial features extracted by the convolutional and pooling layers using them to make predictions. This layer is used at the end of the proposed CNN to map the learned features into the final output, such as class scores. Mathematically, a fully connected layer uses n neurons to receive information from the preceding layer. Each neuron in the FC layers applies a weighted sum of the input values followed by an activation function to generate its output as described in Equation (4) [30]:

(5) $$y_{i}=activation\left(\sum_{j=1}^{n}w_{ij}.x_{i}+b_{i)}\right)$$

where y_i_ is the output of the ith neuron of the FC layer, w_ij_ is the weight connecting the jth neuron in the previous layer to the ith neuron in the fully connected layer, x_j_ represents the output of the jth neuron from of the prior layer, b_i_ denotes the bias term for the ith neuron in the fully connected layer, and the activation function adds nonlinearity to the neuron’s output.

- Softmax layer: This converts the output of the fully connected layer into a probability distribution, assigning a probability to each class. In our case, the Softmax layer assigns each myocardial sub-segment a hypertrophy score (0, 1, 2, or 3) corresponding to the hypertrophy severity, with 0 indicating no hypertrophy and 3 representing the most severe level. Mathematically, the Softmax function can be expressed as follows [29]:

(6) $$f_{j}(Z)=\frac{e^{z}j}{\sum_{k}e^{z}k}$$

- Dropout layer: In our CNN, the dropout layer serves as regularization technique used to prevent overfitting in neural networks and improve the model’s ability to generalize to new data. It works by randomly deactivating a fraction of neurons during each training iteration, forcing the network to learn more robust features, which results in more reliable classification of myocardial sub-segments. A detailed overview of the architecture is provided in Table 1, outlining the configuration and parameters of each layer.

The automatic classification process of myocardial sub-segments according to hypertrophy severity is described in Algorithm 1.
**Algorithm 1**: Classification algorithm of myocardial sub-segments according to hypertrophy severity ## INPUT 2D cine-MRI dataset; (1) Min-max normalization; (2) Automatic segmentation of left ventricle endocardial and epicardial contours using U-Net; RWT: Regional Wall Thickness; Score = [0,1,2,3] # Scores identifying the severity of hypertrophy; N = 49 #Total number of sub-segments per patient; ## OUTPUT Training(1)Start;(2)Train the proposed CNN architecture on the preprocessed dataset;(3)Compile the model with the following:*Three types of optimizers, which are Adam, AdaMax and RMSprop;*Three values of Learning Rate *(LR)* which are 10^−1^, 10^−2^ and 10^−3^;*Seven values of Number of epochs (E) which are 10, 20, 30, 40, 50, 60,70.(4)Evaluate the training accuracy value and the validation loss value for each step.
Testing
(5)Assess the trained model on the test dataset using the “get_predictions ()” function to generate predictions for the test images;(6)Calculate the accuracy score using the predicted labels and the ground truth labels;(7)Compare the obtained results and choose the hyperparameters: “optimizer, LR and E”;Classification: choose one of the four classes based on the RWT values(8)for i = 1: N do(9)         if RWT < 15 mm(10)        Score ← 0(11)            print (“No hypertrophy”)(12)        else if     RWT ≥ 15 mm & RWT ≤ 20 mm(13)        Score ← 1(14)            print (“Mild hypertrophy”)(15)        else if     RWT > 20 mm & RWT ≤ 25 mm(16)        Score ← 2(17)            print (“Moderate hypertrophy”)(18)        else if     RWT > 25 mm(19)        Score ← 3(20)            print (“Severe hypertrophy”)(21)        end(22)end

### 3.6. Performance Metric Evaluation

The performance of the proposed framework was evaluated using several approaches. Initially, we assessed the accuracy of the left ventricular contour segmentation by calculating the Dice Similarity Coefficient (DSC) and the Hausdorff Distance (HD) expressed as follows [31]:

(7) $$DSC=\frac{2TP}{2TP+FP+FN}\;*\;100$$

(8) $$HD=max(\max p⸦C_{A}\;d\left(p,C_{B}\right),\;\max q⸦C_{B}\;d\left(q,C_{A}\right)$$

where a true positive (TP) occurs when the model successfully identifies a positive class, whereas a true negative (TN) is when it correctly recognizes a negative class. A false positive (FP) occurs when the model erroneously labels a negative class as positive, and a false negative (FN) arises when the model mistakenly classifies a positive class as negative. C_A_ represents the automatic contours and C_B_ denotes the reference contours. The term d(p, C) refers to the shortest distance between a point p and the contour C.

To assess the quantification performance of regional myocardial thickness, we computed the mean absolute error (MAE) between the reference values (y) and the predicted values (ŷ) for all frames, using the following formula [8]:

(9) $$MAE\left(y,ŷ\right)=\frac{1}{N}\sum_{i=1}^{N}\left|y^{i}-{ŷ}^{i}\right|$$

To evaluate the ability of our neural network to accurately classify the myocardial sub-segments according to the severity of hypertrophy, we assessed its performance using several metric coefficients including accuracy, precision, recall, and F1-score [8,29]:

(10) $$Accuracy\left(\%\right)=\frac{\left(TP+TN\right)}{\left(TP+TN+FP+FN\right)}\;*\;100\;$$

(11) $$Precision=\frac{TP}{\left(TP+FP\right)}$$

(12) $$Recall=\frac{TP}{\left(TP+FN\right)}$$

(13) $$F_{1}-score=2\;*\;\frac{Precision\;\cdot \;\;Recall}{Precision+Recall}\;$$

These metrics were computed using the macro-averaging approach, which calculates each score independently for every class and then computes the unweighted mean of the results. This method ensures that all severity levels are equally represented in the final evaluation, regardless of the number of samples in each class. Continuous variables were presented as means ± standard deviations (SDs), while categorical data were summarized as numbers and percentages. A *p*-value of less than 0.05 was considered statistically significant. All statistical analyses were conducted using SPSS Statistics software (version 19; SPSS for Windows).

### 3.7. Expert-Driven Dataset Annotation and Validation Process

The creation of our dataset was a demanding process that required extensive manual annotation by expert radiologists. For each of the 133 patients, annotations were performed twice: first using the standard 17-segment model, resulting in 2261 myocardial segments, and then with the more detailed 49-sub-segment model, totaling 6517 sub-segments. This annotation involved a careful visual examination of cine-MRI sequences and the precise assignment of hypertrophy severity levels to each segment and sub-segment. These specific challenges related to manual annotation, combined with the limited availability of expert radiologists, who also participated in the clinical validation phase, strongly influenced the final dataset size. Expanding the sample while maintaining consistent annotation quality and clinical relevance was not feasible within a reasonable timeframe.

To evaluate the clinical impact of the proposed method, we integrated it into a realistic diagnostic workflow. In a two-step validation process, expert radiologists first conducted an independent assessment without any algorithmic support. One week later, they repeated the analysis with access to the automated classification results, which were visualized as bull’s-eye diagrams to assist in their clinical judgment. This validation was performed on the test dataset comprising 27 patients, totaling 1323 sub-segments. Inter- and intra-observer variability were then measured to assess how the integration of our method influenced expert decision-making. This approach provided a robust evaluation of the model’s potential utility under practical clinical conditions.

## 4. Experiments and Results

### 4.1. Hyperparameters Tuning

The choice of hyperparameters depends on the model architecture, dataset characteristics, and the problem being addressed. Therefore, careful experimentation is required to identify the most effective combination of these hyperparameters for a specific task in order to achieve optimal model performance. In this study, the proposed model was initially trained using three optimizers including RMSprop, Adam, and Adamax to identify the most suitable optimizer for our neural network. We tested three mini-batch sizes: 4, 8, and 16. However, the size of 16 was found to be too noisy and required excessive memory resources. We also evaluated the model’s performance across different learning rate values (10^−1^, 10^−2^, 10^−3^).

During the training process, we continuously monitored the training accuracy and validation loss at each iteration. The training process was carried out until the training accuracy ceased to improve and the validation loss reached its minimum value. Once this was accomplished, the required number of epochs for model convergence was recorded. Subsequently, hyperparameters were refined using the validation dataset and the final evaluation metrics were computed based on the test dataset. The proposed classification model was implemented using the Python programming language (version 3.6) on a system equipped with an Intel Core i7 central processing unit (CPU) and 64 GB of RAM. The results are outlined in Table 2.

According to the results shown in Table 2, the best classification performance was achieved with the Adamax optimizer using a learning rate of 10^−3^ and a mini-batch size of 4 across 10, 20, 30, 40, 50, 60, and 70 epochs, which were sufficient for convergence (best performance values are presented in bold and italics (Table 2). The superior performance of Adamax compared to Adam and RMSprop can be attributed to several key characteristics. Firstly, Adamax uses the infinity norm for adaptive learning rates, which enhances stability and effectively manages large gradients. Additionally, it is more robust to outliers, making it well-suited for noisy datasets. Finally, Adamax efficiently tracks maximum past gradients, ensuring stable updates even in the presence of varying magnitudes.

**Figure 4:**
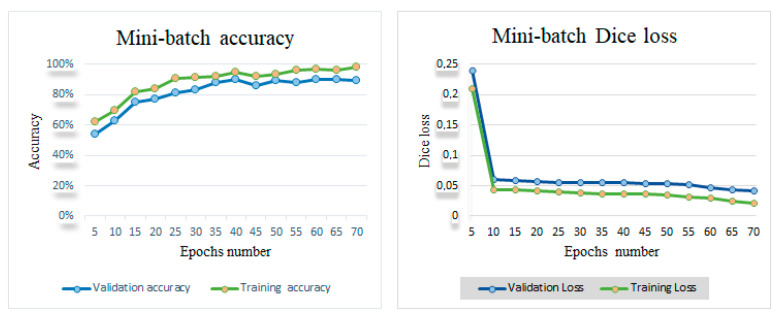
Mini-batch accuracy and Dice loss during the training and validation phase.

Figure 4 illustrates the evolution of mini-batch accuracy and Dice loss function over training and validation epochs. As shown in Figure 4, the accuracy varied from 62% to 98.10% during training and from 54% to 97.90% during validation. Regarding the loss function, it decreases from 0.21 to 0.021 during training and from 0.24 to 0.041 during validation.

Once the optimal configuration of our convolutional neural network was identified using the conventional max pooling operation, considering the number of layers, learning rate, optimizer, and batch size, we conducted an additional evaluation to assess the impact of the pooling strategy. For this purpose, max pooling was replaced by average pooling, while all other architectural components remained unchanged. The comparative performance outcomes of both pooling strategies are summarized in Table 3.

As shown in Table 3, the use of average pooling yielded slightly higher performance across all key evaluation metrics with an accuracy of 98.19%, a precision of 98.27%, a recall of 99.23%, and an F1-score of 98.70%, compared to max pooling. This consistent improvement indicates that average pooling can offer a more balanced feature representation by integrating all spatial activations within the pooling window, which may help preserve subtle structural variations that are clinically relevant in the context of hypertrophy classification. While max pooling is effective in highlighting the most prominent features, it may underrepresent fine details that are critical for detecting different levels of myocardial wall thickening. It is worth noting that these results reflect the specific experimental conditions and dataset characteristics associated with our study. The observed advantage of average pooling reinforces its suitability for our application, particularly in distinguishing nuanced morphological differences across myocardial regions affected by varying degrees of hypertrophy.

### 4.2. Results of Automatic Left Ventricle Contours’ Delineation

In this subsection, we present a comparative evaluation of U-Net architectures with different depths (3, 4, and 5 blocks) for the automatic delineation of LV endocardial and epicardial contours. The assessment is based on key segmentation metrics, including the DSC and HD, to quantify both overlap accuracy and boundary precision. This analysis aims to determine the most effective architecture for accurate and generalizable segmentation.

As mentioned in Section 3.3.2, we initially implemented the original U-Net design with five encoder–decoder blocks, following the configuration proposed by Ronneberger et al. [27]. However, during training on our relatively small dataset (133 patients), we observed perfect training accuracy (100% DSC), alongside a noticeable performance drop on the validation (96.5%) and test sets (96.4%) as presented in Table 4. This significant gap (>3%) between training and generalization performance, combined with an increasing validation loss, further supports the hypothesis of overfitting.

According to the results presented in Table 4, the 3-block U-Net demonstrated limited learning capacity, as reflected by lower DSC scores across all data subsets. In contrast, the 4-block model achieved a better balance, with high training performance (98.1%) and nearly identical validation (97.9%) and test (97.85%) results, demonstrating superior generalization.

To better assess the impact of network depth, we evaluated the segmentation performance of different U-Net architectures using DSC and HD metrics for both endocardial and epicardial contours in healthy individuals and patients with HCM. Results are summarized in Table 5.

Based on the results presented in Table 5, the 4-block architecture demonstrates the most balanced and reliable performance across all contour types and subject groups. Although the 5-block model shows slightly higher DSC values for healthy subjects, it also exhibits higher variability and increased HD, especially in the HCM cases. Importantly, since the training set included both healthy individuals and patients with HCM, these differences do not reflect group-based generalization issues but rather the model’s overall ability to generalize to unseen data. The 4-block configuration maintains high accuracy and low HD consistently, confirming it as the optimal architecture and supporting the hypothesis of overfitting with deeper networks as observed in Table 4. Together, these results justify our selection of the 4-block U-Net architecture as the best compromise between model complexity and generalization capability.

The analysis of Table 5 highlights the strong performance of the 4-block U-Net architecture, which achieved a DSC of up to 99.23% and a HD of 4.103 ± 1.4 mm for epicardial segmentation in healthy subjects. In patients with HCM, the model maintained a high level of accuracy, with a DSC of 98.47% and an HD of 5.424 ± 2.2 mm, demonstrating consistent and reliable segmentation performance across subject groups.

When comparing segmentation accuracy between the epicardial and endocardial contours, it becomes evident that the epicardial boundary is delineated with greater precision. This discrepancy may be attributed to the presence of papillary muscles and trabeculae, which exhibit intensity profiles similar to that of the endocardium, thereby increasing the likelihood of boundary misclassification during endocardial segmentation.

### 4.3. Outcomes of Automated Regional Wall Thickness Quantification

In this subsection, we present in Table 6 the myocardial thickness quantification results for 49 sub-segments in a patient with HCM, along with the corresponding MAE. The segments affected by hypertrophy are highlighted in bold to indicate their thickness values.

The analysis of the obtained results demonstrates that the proposed method enables precise, regional quantification of myocardial thickness compared to the clinically routine method, which relies on semi-automated AHA segmentation. Specifically, this approach can differentiate healthy sub-segments, those with mild hypertrophy, and those with moderate hypertrophy within the same segment, as observed in segments 7, 1, and 16. In contrast, the conventional method provides a global analysis that can overlook subtle variations in myocardial thickness, leaving the radiologist without detailed sub-segment information. Therefore, the proposed method offers significant advantages by detecting varying degrees of hypertrophy within myocardial segments, with a minimal MAE of 1.01 ± 1.16. The highest errors were observed in the apical region, which are likely attributed to the lower resolution of the MRI in this area.

**Figure 5:**
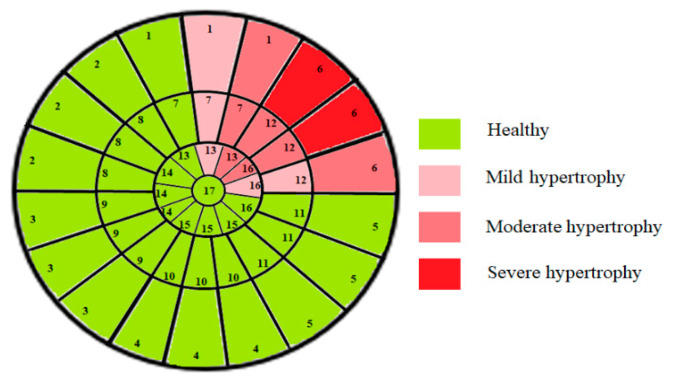
Bull’s-eye diagram classifying the sub-segments into four categories based on the severity of hypertrophy.

This limitation can impact the accuracy of automatic segmentation, which in turn affects the quantification process. For the same patient, we provide a bull’s-eye diagram that organizes all myocardial sub-segments, with each segment color-coded according to hypertrophy severity to enhance interpretability (Figure 5).

### 4.4. Clinical Relevance of the Proposed Approach for Classifying Myocardial Segments by Hypertrophy Severity

In this subsection, we compare the classification performance of the 49-sub-segment model and the standard 17-segment AHA model across different hypertrophy severity classes. The results are summarized in Table 7.

A comprehensive analysis of the results presented in Table 7 demonstrates that the proposed 49-sub-segment model consistently outperforms the conventional 17-segment AHA model across all hypertrophy severity classes. Notably, the 49-sub-segment model achieved superior performance, with accuracy reaching 98.86%, precision 98.79%, recall 99.36%, and F_1_-score 98.91% for the normal class. Similarly high scores were observed for the severe hypertrophy class, with accuracy of 98.71%, precision 98.39%, recall 98.91%, and F_1_-score 98.58%. These findings indicate a more reliable and nuanced classification capability.

This improved performance can be attributed to the underlying methodological differences. The 49-sub-segment model is based on a fully automated segmentation of the endocardial and epicardial contours, allowing for fine-grained and objective quantification of MWT. This enhanced spatial resolution directly benefits the classification process by capturing subtle regional variations associated with different grades of hypertrophy. In contrast, the 17-segment model relies on manually delineated contours and a semi-automatic segmentation of the LV cavity guided by anatomical landmarks, as per the AHA framework. This process is more prone to inter-operator variability and may introduce imprecision, particularly in identifying localized abnormalities. Consequently, the lower spatial granularity and possible inconsistencies inherent in the 17-segment model may compromise the accuracy of hypertrophy classification. Another important observation from the comparison is that, compared to the conventional 17-segment model, our proposed 49-sub-segment model demonstrated significantly improved performance particularly in the classification of myocardial segments with mild and moderate hypertrophy. In contrast, the 17-segment model tended to misclassify mild hypertrophy cases as normal, and moderate cases as severe, reflecting a reduced ability to distinguish between adjacent severity levels. This discrepancy can be attributed to the resolution of thickness representation in each model.

Furthermore, the 49-sub-segment approach assigns three distinct thickness measurements to each AHA segment by dividing it into smaller angular regions, thereby capturing subtle intra-segmental variations. Conversely, the standard 17-segment model provides only a single wall thickness value per segment, covering 60° in basal and mid-ventricular slices and 90° in apical slices, which may obscure finer structural changes. Consequently, the lower granularity of the 17-segment model may limit its sensitivity to the gradual anatomical changes’ characteristic of intermediate stages of hypertrophy, thereby affecting its classification accuracy in these categories. In contrast, our model’s finer segmentation provides a more detailed representation of MWT, enabling improved differentiation across all levels of hypertrophy severity.

**Figure 6:**
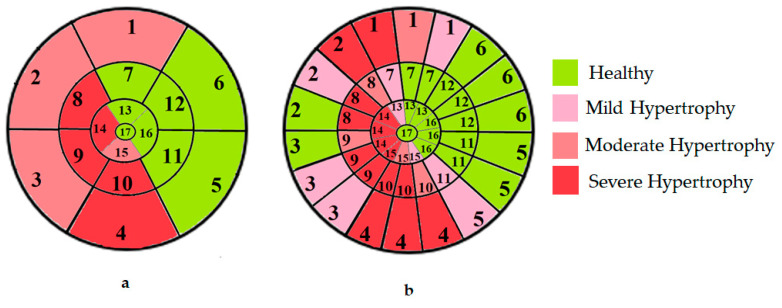
Visualization of myocardial sub-segments in a bull’s-eye diagram in a patient with multiple hypertrophied segments in both 17-segment model (**a**) and 49-sub-segment model (**b**).

To further evaluate the impact of the proposed 49-sub-segment model on classification performance, Table 8 summarizes the classification outcomes at both segmental and sub-segmental levels for a patient with multiple hypertrophied segments, across the basal, mid, and apical slices, comparing the standard 17-segment model and the proposed 49-sub-segment model. To complement these quantitative results and better illustrate the potential clinical impact of the enhanced spatial resolution, we include bull’s-eye diagrams for both models (Figure 6). These visualizations provide an intuitive understanding of how the 49-sub-segment approach may improve the localization and characterization of myocardial abnormalities in such complex clinical cases.

The classification performance illustrated in Table 8 and Figure 6 clearly highlights the added value of the proposed 49-sub-segment model over the conventional 17-segment model, particularly in complex clinical scenarios involving multiple hypertrophied regions. Quantitative analysis shows that the 17-segment model results in a total of 17 misclassifications, distributed as follows: 9 in the basal slice, 5 in the mid-ventricular slice, and 3 in the apical slice. These errors predominantly involve mildly hypertrophied regions that were either misclassified as healthy or segments for which an incorrect hypertrophy severity level was assigned. This misclassification pattern suggests a lack of sensitivity in the 17-segment model when dealing with subtle or early-stage hypertrophic changes. In contrast, there was full agreement between both models regarding the classification of the apex.

More importantly, a qualitative examination of the bull’s-eye diagrams reveals the diagnostic advantage of the finer anatomical resolution. In the 17-segment model (Figure 6a), the hypertrophy classification is often generalized across large segments, potentially masking the presence of mild or moderate hypertrophy within a single segment. In contrast, the 49-sub-segment model (Figure 6b) captures more nuanced regional variations, allowing for a more accurate spatial delineation of the severity levels. For instance, in the basal and mid slices, several sub-segments within a single 17-segment region display different degrees of hypertrophy (mild and moderate), which this model fails to detect.

To provide a more comprehensive assessment of the proposed model’s performance across varying clinical presentations, Table 9 presents classification outcomes for three additional patients, each with localized hypertrophy affecting different cardiac regions.

Table 9 further supports the clinical relevance of the proposed 49-sub-segment model by presenting classification results for three patients with regionally localized hypertrophy. Across all three cases, the bull’s-eye diagrams reveal that the proposed model provides a more detailed representation of hypertrophy distribution, capturing spatial heterogeneity that remains undetected by the conventional 17-segment model. Notably, in the case of patient 2, the 49-sub-segment model successfully identified a mild hypertrophy that was missed by the 17-segment model, highlighting the model’s ability to detect early-stage or subtle myocardial alterations with greater sensitivity. These findings reinforce the value of the proposed model in delivering more precise spatial characterization of myocardial hypertrophy across various anatomical locations.

To assess the clinical applicability and generalizability of our method, we applied it to the test dataset following the protocol outlined in Section 3.7. This step aimed to evaluate its practical performance in realistic scenarios and its potential integration into routine clinical workflows. The analysis of 1323 sub-segments from the 27 patients involved in the clinical validation phase resulted in the findings presented in Table 10. The reference diagnosis for both the visually established diagnosis and the one based on quantitative results is the diagnosis collaboratively established by the two radiologists.

As presented in Table 10, the quantitative approach, which emphasizes accurate regional quantification of myocardial thickness, demonstrated significantly superior performance compared to visual analysis. It achieved an accuracy of 98.19%, a precision of 98.27%, a recall of 99.13%, and an F1-score of 98.7%. Regarding intra-observer variability, radiologist 1 (R1) exhibited a rate of 6.72%, while radiologist 2 (R2) achieved a lower variability of 2.88%. This difference may be attributed to the additional four years of experience held by R2, which likely contribute to a more consistent assessment. The integration of our method into clinical practice notably reduced inter-observer variability from 6.04% to 2.19% and minimized both false positives and false negatives.

## 5. Discussion

In this study, we proposed an automated framework to detect, quantify, and assess the severity and extent of LVH using a deep learning-based algorithm involving a CNN. The proposed method focuses on the automatic quantification of regional wall thickness based on contours’ segmentation and LV cavity division into 49 sub-segments according to AHA standards. In this section, we compare the performance of our method with existing approaches for classifying LVH. However, it is important to highlight a number of key points before interpreting this comparison.

First, our method was evaluated on a dedicated dataset specifically developed for the sub-segmental classification of hypertrophy severity, whereas the majority of existing studies were conducted on different datasets, with distinct clinical objectives and annotation protocols. Most of these studies focus on identifying the etiology of LVH such as distinguishing between HCM and hypertensive heart disease rather than quantifying the degree of hypertrophy at a regional level.

Second, the variability in patient populations, imaging acquisition settings, and classification criteria introduces significant heterogeneity across studies, making direct comparison of performance metrics difficult. Such differences can introduce variability in performance metrics across studies, underscoring the importance of interpreting these results within the methodological and clinical context in which each model was developed and tested.

Despite these challenges, it remains important to situate our work within the broader landscape of LVH classification research. Accordingly, Table 11 presents a selection of studies closely aligned with our clinical and methodological context. Although these works were not validated on the same dataset, they provide informative reference points and contextual benchmarks that highlight both the originality of our approach and its potential clinical relevance.

In Table 11 we present a selection of recent studies addressing LVH classification using a range of imaging modalities and datasets. These studies were conducted on varying population sizes, from institutional cohorts, such as NIH databases, to independent patient populations with sample sizes ranging from 300 to over 1000 cases. Such diversity in datasets, along with differences in clinical focus and imaging modalities, naturally impacts the reported performance metrics. The proposed method in this study was applied to a dataset comprising 133 patients, with each patient represented by approximately 20 to 40 short-axis cine-MRI slices, resulting in a total of more than 3325 images.

Although moderate in size, this dataset enables a detailed sub-segmental analysis of myocardial hypertrophy severity, which clearly distinguishes our work from most existing studies. In effect, despite differences in datasets and evaluation criteria, our method demonstrates competitive performance, achieving an accuracy of 98.19%, a precision of 98.27%, a recall of 99.13%, and an F1-score of 98.7%. These results position the proposed approach favorably compared to several referenced methods [3,32,34,35,36,37,38], particularly among those relying on cine-MRI data.

On the other hand, it is important to note that most methods based on X-ray datasets included in this comparison exhibit lower classification performance than those relying on MRI data. This difference can be attributed to the nature of radiographic imaging, which often focuses on global cardiac features such as myocardial volume or the heart’s position within the thoracic cavity [39]. These approaches tend to overlook the internal structure of the myocardium and its regional characteristics, thus limiting their ability to provide accurate assessments of LVH.

In contrast, MRI offers superior spatial resolution and tissue contrast, making it particularly well-suited for detailed cardiac assessment. These advantages are reflected in the generally higher performance metrics observed in MRI-based studies. Among them, the approach proposed by Wu et al. [33] achieved an accuracy of 98.40% and a recall of 99.2%, slightly surpassing the performance of our model in those specific metrics. However, our method demonstrated a higher precision (98.27%) and F1-score (98.7%), highlighting the effectiveness of our classification framework. Overall, these observations support the use of MRI as a preferred imaging modality for the diagnosis of LVH. Its spatial resolution enables more robust, reliable, and anatomically detailed analysis, particularly when compared to other imaging modalities such as echocardiography [40,41,42].

In the study by Budai et al. [3], an automatic model was proposed to identify left ventricular hypertrophy using cine cardiac MR images in both short- and long-axis views. In contrast, our method offers a more refined deep learning framework that classifies 49 myocardial sub-segments by hypertrophy severity, based on subtle variations in wall thickness. This granularity enables precise regional analysis and a comprehensive assessment of ventricular remodeling.

In terms of performance, our method outperformed the model proposed in [3], which achieved 91% precision, 97% recall, and 92% F1-score. This difference may be partly attributed to architectural choices. The authors in [3] employed a 3D ResNet model, which exhibited slower training and suboptimal performance on short-axis views. To address these issues, they increased the network complexity by expanding it to 16 convolutional layers and incorporating 8 residual blocks. In contrast, our method adopted a simpler yet effective CNN architecture consisting of five convolutional layers, trained in just 3 h and 21 min and demonstrating competitive performance.

The proposed method offers a comprehensive framework for the detailed assessment of LVH, addressing both the spatial extent and severity of myocardial involvement. Through the integration of RWT quantification and effective classification techniques, the method enables highly detailed identification and grading of hypertrophic regions, significantly enhancing the precision of cardiac assessment. Clinically, this refined regionality supports more targeted and informed decision-making by providing radiologists with actionable insights on the exact location and extent of hypertrophy. Consequently, the proposed approach can guide treatment plans tailored to individual patient needs, improve monitoring of disease progression, and facilitate more precise risk stratification in clinical practice.

**Limitations and future directions:**

While the proposed method demonstrates promising performance and clinical relevance, certain limitations should be acknowledged to guide future improvements and extensions. First, the model was trained and validated exclusively on short-axis cine-MRI views from a relatively small cohort of 133 patients. Extending the approach to incorporate additional imaging planes, such as two-, three- and four-chamber views, will likely improve classification accuracy in challenging regions like the apex, where spatial resolution is limited. In this study, we excluded healthy athletes from our dataset, although it is well established that LVH can occur as a physiological adaptation in athletic hearts. Future work should therefore aim to distinguish pathological hypertrophy from these physiological forms, a clinically important differentiation that could improve diagnostic accuracy. Lastly, the method was compared against approaches not validated on the same datasets, primarily due to the lack of available methods trained on comparable databases, which necessitates cautious interpretation of the comparative results and underscores the need for standardized benchmarks in future evaluations.

## 6. Conclusions

In this study, we developed an automatic and fine-grained framework for the assessment of LVH, using a CNN trained to classify myocardial regions according to a novel 49-sub-segment model. Each sub-segment is categorized into one of four severity levels of hypertrophy, enabling a detailed and region-specific evaluation based on automated wall thickness measurements. The proposed method demonstrated high performance, achieving an accuracy of 98.19%, a precision of 98.27%, a recall of 99.13%, and an F1-score of 98.7%, reflecting its ability to accurately capture subtle anatomical variations and disease severity. These robust results underline the reliability of our approach and emphasize its strong potential for integration into clinical workflows, where accurate and spatially resolved assessment of LVH plays a critical role in patient care.

## Figures and Tables

**Table 1:** Detailed information on the CNN model tailored for sub-segment classification according to hypertrophy severity (BN: batch normalization, FC: fully connected).

Layer Name	Layer Type	Kernal Size	Filters	Stride	Padding	Activation	Output Shape	Parameters
1. Input	Input	-	-	-	-	-	256 × 256 × 3	0
2. Convolutional	Conv 2D	3 × 3	32	1 × 1	Same	-	256 × 256 × 32	896
3. Activation layer	Activation	-	-	-	-	ReLU	256 × 256 × 32	0
4. BN	Batch normalization	-	-	-	-	-	256 × 256 × 32	64
5. Average pooling	Average pooling 2D	2 × 2	-	2 × 2	-	-	128 × 128 × 32	0
6. Convolutional	Conv 2D	3 × 3	64	1 × 1	2	-	130 × 130 × 64	18,496
7. Activation layer	Activation	-	-	-	-	ReLU	130 × 130 × 64	0
8. BN	Batch normalization	-	-	-	-	-	130 × 130 × 64	128
9. Average pooling	Average pooling 2D	2 × 2	-	2 × 2	-	-	65 × 65 × 64	0
10. Convolutional	Conv 2D	3 × 3	128	1 × 1	2	-	67 × 67 × 128	73,856
11. Activation	Activation	-	-	-	-	ReLU	67 × 67 × 128	0
12. BN	Batch normalization	-	-	-	-	-	67 × 67 × 128	256
13. Average pooling	Average pooling 2D	2 × 2	-	2 × 2	-	-	33 × 33 × 128	0
14. Convolutional	Conv 2D	3 × 3	256	1 × 1	2	-	35 × 35 × 256	295,168
15. Activation	Activation	-	-	-	-	ReLU	35 × 35 × 256	0
16. BN	Batch normalization	-	-	-	-	-	35 × 35 × 256	512
17. Average pooling	Average pooling 2D	2 × 2	-	2 × 2	-	-	17 × 17 × 256	0
18. Convolutional	Conv 2D	3 × 3	512	1 × 1	2	-	19 × 19 × 512	1,181,160
19. Activation	Activation	-	-	-	-	ReLU	19 × 19 × 512	0
20. BN	Batch normalization	-	-	-	-	-	19 × 19 × 512	1024
21. Average pooling	Average pooling 2D	2 × 2	-	2 × 2	-	-	9 ×9 ×512	0
22. FC layer	Dense 1	-	-	-	-	ReLU	128	5,308,544
23. Dropout layer	Dropout	Dropout rate = 0.3					128	0
24. FC layer	Dense 2	-	-	-	-	ReLU	16	2064
25. Classification	Softmax classifier	-	-	-	-	Softmax	4	0

**Table 2:** Comparison of model performance by optimizer with mini-batch sizes 4 and 8 and different learning rates.

Parameters		Epochs	Batch-Size = 4			Batch-Size = 8		
			Rmsprop	Adam	Adamax	Rmsprop	Adam	Adamax
LR	10^−1^	10	51.43	53.12	54.34	49.44	52.67	52.61
		20	54.67	56.24	58.63	52.09	53.18	54.29
		30	61.44	63.71	68.92	58.25	62.14	63.48
		40	63.56	67.81	70.41	62.15	66.43	68.17
		50	70.91	74.26	82.74	68.22	71.61	77.02
		60	76.13	81.67	87.15	73.43	79.25	82.45
		70	81.07	90.24	91.02	79.16	82.77	89.27
	10^−2^	10	52.14	53.99	54.67	51.32	52.89	53.23
		20	55.21	57.71	61.23	53.15	54.04	57.51
		30	62.17	65.04	71.56	60.43	63.38	69.27
		40	70.63	72.31	86.45	64.52	70.12	81.13
		50	80.06	84.22	90.28	78.16	81.36	87.71
		60	83.22	87.63	92.64	80.72	83.23	91.44
		70	84.46	92.58	94.72	82.83	84.37	93.10
	10^−3^	10	56.73	59.28	69.82	53.44	56.69	64.32
		20	59.40	61.13	74.56	56.03	58.23	60.17
		30	64.31	72.23	86.33	60.38	64.78	70.42
		40	73.60	83.88	91.56	71.04	80.47	86.65
		50	86.44	87.21	93.03	82.56	85.09	89.21
		60	90.45	92.19	96.72	87.22	89.13	91.52
		70	92.03	94.15	98.10	90.82	92.62	94.63

**Table 3:** Comparison of average and max pooling performance in CNN-based classification of cardiac hypertrophy severity.

Metric	Average Pooling (Selected)	Max Pooling
Accuracy (%)	98.19	97.41
Precision (%)	98.27	97.33
Recall (%)	99.23	98.02
F_1_-Score (%)	98.70	97.67

**Table 4:** Segmentation performance in terms of (DSC %) of U-Net architectures across training, validation, and test sets for different block depths.

Number of Blocks	Training DSC (%)	Validation DSC (%)	Test DSC (%)	Observation
3	96.80	95.5	95.70	Underfitting
4 (Selected)	98.10	97.90	97.85	Best overall performance
5	100	96.50	96.40	Overfitting suspected

**Table 5:** Segmentation performance comparison of U-Net architectures with varying depths on endocardial (endo) and epicardial (epi) contours in healthy and HCM subjects, evaluated by DSC and HD metrics.

Metric	Group	3 Blocks	4 Blocks (Selected)	5 Blocks
Mean DSC (%)	Endo-Healthy	96.32 ± 3.5	98.05 ± 2.8	98.40 ± 1.9
	Endo-HCM	95.90 ± 2.7	97.68 ± 1.6	97.20 ± 3.4
	Epi-Healthy	97.78 ± 4.5	99.23 ± 3.1	99.50 ± 2.2
	Epi-HCM	97.10 ± 5.0	98.47 ± 4.2	97.90 ± 5.8
Mean HD (mm)	Endo-Healthy	6.89 ± 3.1	5.678 ± 2.6	5.45 ± 2.2
	Endo-HCM	7.45 ± 3.9	6.345 ± 3.5	6.90 ± 3.8
	Epi-Healthy	5.91 ± 2.6	4.103 ± 1.4	4.00 ± 1.3
	Epi-HCM	6.39 ± 2.8	5.424 ± 2.2	5.90 ± 2.9

**Table 6:** Myocardial thickness quantification results and MAE by sub-segment for a patient with HCM.

Myocardial Section	Myocardial Segments	Mean Thickness Values for All Sub-Segments (mm)			MAE		
		1st	2nd	3rd	1st	2nd	3rd
Basal	1	**21.23**	**18.12**	14.78	1.23 ± 0.5	1.44 ± 1.1	1.07 ± 1.3
	2	7.84	7.63	6.51	1.42 ± 1.01	1.42 ± 1.02	1.21 ± 2.4
	3	6.57	7.2	6.22	1.18 ± 3.22	1.20 ± 1.23	1.25 ± 2.3
	4	8.2	10.44	7.99	1.32 ± 2.5	1.11 ± 2.91	1.24 ± 1.15
	5	9.32	12.67	8.71	1.24 ± 1.04	1.22 ± 1.01	1.22 ± 1.20
	6	**25.61**	**26.08**	**22.77**	1.01 ± 1.16	1.19 ± 0.81	1.23 ± 1.12
Median	7	12.44	**16.08**	**22.08**	1.15 ± 1.12	1.27 ± 1.02	1.09 ± 1.22
	8	11.50	10.34	9.17	1.03 ± 1.37	1.13 ± 1.04	1.21 ± 1.41
	9	13.21	11.72	11.13	1.31 ± 1.07	1.05 ± 1.22	1.28 ± 1.09
	10	13.82	13.40	12.61	1.27 ± 1.15	1.28 ± 0.87	1.25 ± 1.13
	11	12.47	13.12	14.55	1.23 ± 1.41	1.31 ± 1.09	1.13 ± 1.02
	12	**23.54**	**21.91**	**15.89**	1.23 ± 1.03	1.22. ±0.81	1.02 ± 0.43
Apical	13	14.56	**16.34**	**21.61**	1.41 ± 1.2	1.28 ± 1.03	1.33 ± 0.98
	14	12.28	13.49	13.01	1.29 ± 0.87	1.39 ± 1.52	1.28 ± 0.73
	15	9.13	10.32	10.75	1.39 ± 1.47	1.50 ± 1.31	1.28 ± 1.06
	16	11.93	**16.61**	**21.18**	1.37 ± 0.88	1.37 ± 0.9	1.42 ± 0.48
Apex	17	5.31			1.28 ± 1.34		

**Table 7:** Comparison of classification performance between the 49-sub-segment and 17-segment AHA models across hypertrophy severity levels.

Hypertrophy Class	Model	Accuracy (%)	Precision (%)	Recall (%)	F_1_-Score (%)
Normal	17-segment	95.42	94.81	95.92	96.22
	49-sub-segment	98.86	98.79	99.36	98.91
Mild Hypertrophy	17-segment	91.27	90.72	87.54	91.46
	49-sub-segment	95.92	96.81	95.91	96.55
Moderate Hypertrophy	17-segment	90.33	89.47	85.43	89.67
	49-sub-segment	94.54	96.96	94.18	97.14
Severe Hypertrophy	17-segment	94.66	94.12	94.17	94.62
	49-sub-segment	98.71	98.39	98.91	98.58
Average (All classes)	17-segment	92.92	92.28	90.76	92.99
	49-sub-segment	97.01	97.73	97.09	97.80

**Table 8:** Classification results of myocardial segments and sub-segments in a patient with multiple hypertrophied segments using the 17-segment and 49-sub-segment models.

Section	Segmented Cine-MRI	17-Segment Model	Proposed 49-Sub-Segment Model	Number of Misclassifications
Basal section	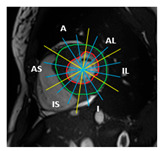	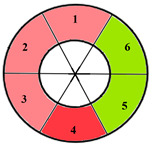	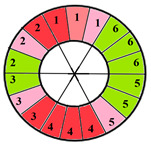	9 sub-segments (2 sub-segments of segment 1 + 3 sub-segments of segment 2 + 3 sub-segments of segment 3 + 1 sub-segment of segment 5)
Mid-section	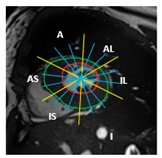	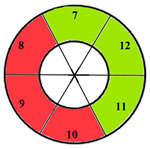	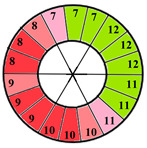	5 sub-segments (1 sub-segment of segment 7 + 1 sub-segment of segment 8 + 1 sub-segment of segment 9 + 1 sub-segment of segment 10 + 1 sub-segment of segment 11)
Apical section	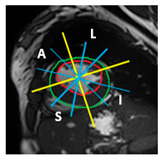	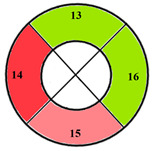	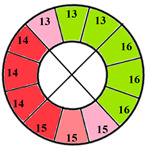	3 sub-segments (1 sub-segment of segment 13 + 2 sub-segments of segment 15)

**Table 9:** Classification results in patients with regionally localized hypertrophy: comparison between 17- and 49-sub-segment models.

Patients	17-Segment Model	49-Sub-Segment Model	Number of Misclassifications
Patient 1	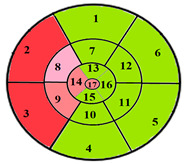	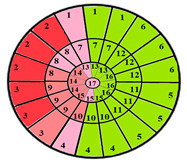	9 sub-segments
Patient 2	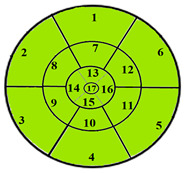	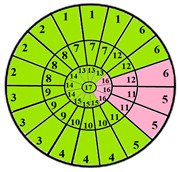	9 sub-segments
Patient 3	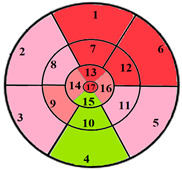	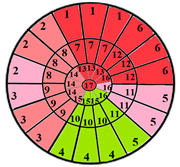	13 sub-segments

**Table 10:** Performance measures for visual and quantitative diagnoses in terms of accuracy (Acc%), precision (Pre%), recall (Re%), F1-score (%), and intra- and inter-observer variability (%).

	Performance Measures										Intra-Observer Variability
	Visual Analysis					Quantitative Analysis					
	Results	Acc	Pre	Re	F1-Score	Results	Acc	Pre	Re	F1-score	
R1	TP = 827 TN = 354 FP = 77 FN = 65	89.27	91.48	92.71	92	TP = 847 TN = 423 FP = 32 FN = 21	95.99	96.36	97.58	97	6.72%
R2	TP = 881 TN = 380 FP = 35 FN = 27	95.31	96.18	97.03	96.6	TP = 910 TN = 389 FP = 16 FN = 8	98.19	98.27	99.13	98.7	2.88%
Inter-observer variability	6.04%					2.19%					-

**Table 11:** Comparison of the proposed method to the state-of-the art methods.

**Reference**	**Year**	**Database**	**Performance Metrics**			
			**Accuracy**	**Precision**	**Recall**	**F_1_-Score**
Budai et al. [3]	2022	CMR dataset 428 patients + 234 healthy subjects	-	91	97	92
Lim et al. [32]	2022	300 patients	98.00	97.80	98.20	97.99
Wu et al. [33]	2022	National institutes of health (NIH) database	98.40	97.60	99.20	98.38
Chen et al. [34]	2022	NIH	90.50	-	94.45	90.59
Ajmera et al. [35]	2022	1012 posteroanterior CXRs	-	99.00	80.00	88.00
Ribeiro et al. [36]	2023	VinDr-CXR	91.80	74.00	87.00	79.80
Innat et al. [37]	2023	NIH	87.00	-	85.00	86.00
E. Sorour et al. [29]	2023	NIH	95.50	96.89	73.10	83.30
Peng et al. [38]	2024	442 patients	-	90.5	90.5	90.4
Proposed	2025	133 patients	98.19	98.27	99.13	98.7

## Data Availability

Data are contained within the article.

## Abbreviations

The following abbreviations are used in this manuscript:

AHA	American Heart Association
AI	Artificial Intelligence
CMRI	Cardiac Magnetic Resonance Imaging
CNN	Convolutional Neural Network
CVD	Cardiovascular Disease
DSC	Dice Similarity Coefficient
ECG	Electrocardiogram
HD	Hausdorff Distance
LV	Left Ventricle
LVH	Left Ventricle Hypertrophy
LVM	Left Ventricle Mass
MAE	Mean Absolute Error
RWT	Regional Wall Thickness

## References

[1] Roth G.A., Mensah G.A., Johnson C.O., Addolorato G., Ammirati E., Baddour L.M., Barengo N.C., Beaton A.Z., Benjamin E.J., Benziger C.P. (2020). Global Burden of Cardiovascular Diseases Writing Group. Global burden of cardiovascular diseases and risk factors, 1990–2019: Update from the GBD 2019 study. J. Am. Coll. Cardiol..

[2] Baptista E.A., Queiroz B.L. (2022). Spatial analysis of cardiovascular mortality and associated factors around the world. BMC Public Health.

[3] Budai A., Suhai F.I., Csorba K., Dohy Z., Szabo L., Merkely B., Vago H. (2022). Automated classification of left ventricular hypertrophy on cardiac mri. Appl. Sci..

[4] Méndez C., Soler R., Rodríguez E., Barriales R., Ochoa J.P., Monserrat L. (2018). Differential diagnosis of thickened myocardium: An illustrative MRI review. Insights Into Imaging.

[5] Parlati A.L.M., Nardi E., Marzano F., Madaudo C., Di Santo M., Cotticelli C., Agizza S., Abbellito G.M., Filardi F.P., Del Giudice M. (2025). Advancing Cardiovascular Diagnostics: The Expanding Role of CMR in Heart Failure and Cardiomyopathies. J. Clin. Med..

[6] Grajewski K.G., Stojanovska J., Ibrahim E.-S.H., Sayyouh M., Attili A. (2020). Left ventricular hypertrophy: Evaluation with cardiac MRI. Curr. Probl. Diagn. Radiol..

[7] Maron B.J., Kragel A.H., Roberts W.C. (1990). Case 883 Sudden Death in Hypertrophic Cardiomyopathy with Normal Left Ventricular Mass. Case Reports in Cardiology: Cardiomyopathy. Br. Heart J..

[8] Baccouch W., Hadidi T., Benameur N., Lahidheb D., Labidi S. (2024). Convolution neural network for Objective Myocardial Viability Assessment based on Regional Wall Thickness Quantification from Cine-MR images. IEEE Access.

[9] Lundin M., Heiberg E., Nordlund D., Gyllenhammar T., Steding-Ehrenborg K., Engblom H., Carlsson M., Atar D., van der Pals J., Erlinge D. (2022). Left ventricular mass and global wall thickness–prognostic utility and characterization of left ventricular hypertrophy. MedRxiv.

[10] Maanja M., Noseworthy P.A., Geske J.B., Ackerman M.J., Arruda-Olson A.M., Ommen S.R., Attia Z.I., Friedman P.A., Siontis K.C. (2022). Tandem deep learning and logistic regression models to optimize hypertrophic cardiomyopathy detection in routine clinical practice. Cardiovasc. Digit. Health J..

[11] Maanja M., Schlegel T.T., Kozor R., Lundin M., Wieslander B., Wong T.C., Schelbert E.B., Ugander M. (2020). The electrical determinants of increased wall thickness and mass in left ventricular hypertrophy. J. Electrocardiol..

[12] Kokubo T., Kodera S., Sawano S., Katsushika S., Nakamoto M., Takeuchi H., Kimura N., Shinohara H., Matsuoka R., Nakanishi K. (2022). Automatic detection of left ventricular dilatation and hypertrophy from electrocardiograms using deep learning. Int. Heart J..

[13] Li S., Feng Z., Xiao C., Wu Y., Ye W., Zhang F. (2022). The establishment of hypertrophic cardiomyopathy diagnosis model via artificial neural network and random decision forest method. Mediat. Inflamm..

[14] Duffy G., Cheng P.P., Yuan N., He B., Kwan A.C., Shun-Shin M.J., Alexander K.M., Ebinger J., Lungren M.P., Rader F. (2022). High-throughput precision phenotyping of left ventricular hypertrophy with cardiovascular deep learning. JAMA Cardiol..

[15] Soto J.T., Hughes J.W., Sanchez P.A., Perez M., Ouyang D., A Ashley E. (2022). Multimodal deep learning enhances diagnostic precision in left ventricular hypertrophy. Eur. Heart J.-Digit. Health.

[16] Jian Z., Wang X., Zhang J., Wang X., Deng Y. (2020). Diagnosis of left ventricular hypertrophy using convolutional neural network. BMC Med. Inform. Decis. Mak..

[17] Hwang I.-C., Choi D., Choi Y.-J., Ju L., Kim M., Hong J.-E., Lee H.-J., Yoon Y.E., Park J.-B., Lee S.-P. (2022). Differential diagnosis of common etiologies of left ventricular hypertrophy using a hybrid CNN-LSTM model. Sci. Rep..

[18] Beneyto M., Ghyaza G., Cariou E., Amar J., Lairez O. (2023). Development and validation of machine learning algorithms to predict posthypertensive origin in left ventricular hypertrophy. Arch. Cardiovasc. Dis..

[19] Gupta A., Harvey C.J., DeBauge A., Shomaji S., Yao Z., Noheria A. (2024). Machine learning to classify left ventricular hy-pertrophy using ECG feature extraction by variational autoencoder. MedRxiv.

[20] Lim D.Y., Sng G., Ho W.H., Hankun W., Sia C.-H., Lee J.S., Shen X., Tan B.Y., Lee E.C., Dalakoti M. (2021). Machine learning versus classical electrocardiographic criteria for echocardiographic left ventricular hypertrophy in a pre-participation cohort. Kardiol. Pol..

[21] Kwon J.-M., Jeon K.-H., Kim H.M., Kim M.J., Lim S.M., Kim K.-H., Song P.S., Park J., Choi R.K., Oh B.-H. (2020). Comparing the performance of artificial intelligence and conventional diagnosis criteria for detecting left ventricular hypertrophy using electrocardiography. EP Eur..

[22] Khurshid S., Friedman S., Pirruccello J.P., Di Achille P., Diamant N., Anderson C.D., Ellinor P.T., Batra P., Ho J.E., Philippakis A.A. (2021). Deep learning to predict cardiac magnetic resonance–derived left ventricular mass and hypertrophy from 12-lead ECGs. Circ. Cardiovasc. Imaging.

[23] Zhou H., Li L., Liu Z., Zhao K., Chen X., Lu M., Yin G., Song L., Zhao S., Zheng H. (2021). Deep learning algorithm to improve hypertrophic cardiomyopathy mutation prediction using cardiac cine images. Eur. Radiol..

[24] Rodríguez-De-Vera J.M., Bernabé G., García J.M., Saura D., González-Carrillo J. (2022). Left ventricular non-compaction cardiomyopathy automatic diagnosis using a deep learning approach. Comput. Methods Programs Biomed..

[25] Henderi H., Wahyuningsih T., Rahwanto E. (2021). Comparison of Min-Max normalization and Z-Score Normalization in the K-nearest neighbor (kNN) Algorithm to Test the Accuracy of Types of Breast Cancer. Int. J. Inform. Inf. Syst..

[26] Baccouch W., Oueslati S., Solaiman B., Lahidheb D., Labidi S. (2023). Automatic Left Ventricle Segmentation from Short-Axis MRI Images Using U-Net with Study of the Papillary Muscles’ Removal Effect. J. Med. Biol. Eng..

[27] Ronneberger O., Fischer P., Brox T. (2015). U-net: Convolutional networks for biomedical image segmentation. Medical Image Computing and Computer-Assisted Intervention–MICCAI 2015: 18th International Conference, Munich, Germany, 5–9 October 2015, Proceedings, Part III 18.

[28] Zhu X., Meng Q., Ding B., Gu L., Yang Y. (2019). Weighted pooling for image recognition of deep convolutional neural networks. Clust. Comput..

[29] Sorour S.E., Wafa A.A., Abohany A.A., Hussien R.M., Rajamohan V. (2024). A Deep Learning System for Detecting Cardiomegaly Disease Based on CXR Image. Int. J. Intell. Syst..

[30] Li Z., Liu F., Yang W., Peng S., Zhou J. (2021). A survey of convolutional neural networks: Analysis, applications, and prospects. IEEE Trans. Neural Netw. Learn. Syst..

[31] Baccouch W., Oueslati S., Solaiman B., Lahidheb D., Labidi S. (2024). Automatic left ventricle volume and mass quantification from 2D cine-MRI: Investigating papillary muscle influence. Med. Eng. Phys..

[32] Lin C.-H., Zhang F.-Z., Wu J.-X., Pai N.-S., Chen P.-Y., Pai C.-C., Kan C.-D. (2022). Posteroanterior chest X-ray image classification with a multilayer 1D convolutional neural network-based classifier for cardiomegaly level screening. Electronics.

[33] Wu J.-X., Pai C.-C., Kan C.-D., Chen P.-Y., Chen W.-L., Lin C.-H. (2022). Chest X-ray image analysis with combining 2D and 1D convolutional neural network based classifier for rapid cardiomegaly screening. IEEE Access.

[34] Chen L., Mao T., Zhang Q. (2022). Identifying cardiomegaly in chest X-rays using dual attention network. Appl. Intell..

[35] Ajmera P., Kharat A., Gupte T., Pant R., Kulkarni V., Duddalwar V., Lamghare P. (2022). Observer performance evaluation of the feasibility of a deep learning model to detect cardiomegaly on chest radiographs. Acta Radiol. Open.

[36] Ribeiro E., Cardenas D.A.C., Krieger J.E., Gutierrez M.A. (2023). Interpretable deep learning model for cardiomegaly detection with chest X-ray images. Anais do XXIII Simp´osio Brasileiro de Computação Aplicada a Saude´.

[37] Innat M., Hossain F., Mader K., Kouzani A.Z. (2023). A convolutional attention mapping deep neural network for classification and localization of cardiomegaly on chest X-rays. Sci. Rep..

[38] Peng B., Li X., Li X., Wang Z., Deng H., Luo X., Yin L., Zhang H. (2024). A Deep Learning-Driven Pipeline for Differentiating Hypertrophic Cardiomyopathy from Cardiac Amyloidosis Using 2D Multi-View Echocardiography. arXiv.

[39] Gomes B., Hedman K., Kuznetsova T., Cauwenberghs N., Hsu D., Kobayashi Y., Ingelsson E., Oxborough D., George K., Salerno M. (2023). Defining left ventricular remodeling using lean body mass allometry: A UK Biobank study. Eur. J. Appl. Physiol..

[40] Sivalokanathan S. (2022). The role of cardiovascular magnetic resonance imaging in the evaluation of hypertrophic cardiomyopathy. Diagnostics.

[41] Bukharovich I., Wengrofsky P., Akivis Y. (2023). Cardiac multimodality imaging in hypertrophic cardiomyopathy: What to look for and when to image. Curr. Cardiol. Rev..

[42] De la Garza-Salazar F., Romero-Ibarguengoitia M.E., Rodriguez-Diaz E.A., Azpiri-Lopez J.R., González-Cantu A., Ab Rahman N.H. (2020). Improvement of electrocardiographic diagnostic accuracy of left ventricular hypertrophy using a Machine Learning approach. PLoS ONE.

